# A Traditional Chinese Medicine, Zhenqi Granule, Potentially Alleviates Dextran Sulfate Sodium-Induced Mouse Colitis Symptoms

**DOI:** 10.3390/biology13060427

**Published:** 2024-06-10

**Authors:** Xiuxiu Qiu, Wentao Luo, Haotian Li, Tingting Li, Yaxue Huang, Qi Huang, Rui Zhou

**Affiliations:** 1 State Key Laboratory of Agricultural Microbiology, College of Veterinary Medicine, Huazhong Agricultural University, Wuhan 430070, China; xiuxiuq@webmail.hzau.edu.cn (X.Q.); luowentao@webmail.hzau.edu.cn (W.L.); lht@webmail.hzau.edu.cn (H.L.); li.tingting@webmail.hzau.edu.cn (T.L.); yaxuehuang@webmail.hzau.edu.cn (Y.H.); 2 International Research Center for Animal Disease, Ministry of Science & Technology of China, Wuhan 430070, China; 3 The Cooperative Innovation Center of Sustainable Pig Production, Wuhan 430070, China

**Keywords:** Zhenqi Granule, Dextran Sulfate Sodium, ulcerative colitis, network pharmacology, inflammation

## Abstract

**Simple Summary:**

Ulcerative colitis significantly impacts the quality of life of individuals. Although several therapeutic drugs are available, their efficacy remains suboptimal. Therefore, developing more effective treatment strategies remains to be of great significance. Traditional Chinese Medicine has attracted interest due to its multiple components, multifaceted targets, and minimal side effects. In this study, we reported that Zhenqi Granule, a Traditional Chinese Medicine preparation, exhibited both preventive and therapeutic efficacy that effectively mitigated the symptoms of Dextran Sulfate Sodium-induced ulcerative colitis in mice. The potential mechanism was further analyzed through network pharmacological analysis, suggesting that the active components of Zhenqi Granule target multiple inflammatory pathways. Our study provides a promising strategy for the prevention and treatment of ulcerative colitis.

**Abstract:**

Ulcerative colitis (UC) is an inflammatory bowel disease that causes chronic inflammation in the large intestine. The etiology of UC is complex and incompletely understood, with potential contributing factors including genetic susceptibility, environmental influences, immune dysregulation, and gut barrier dysfunction. Despite available therapeutic drugs, the suboptimal cure rate for UC emphasizes the necessity of developing novel therapeutics. Traditional Chinese Medicine (TCM) has attracted great interest in the treatment of such chronic inflammatory diseases due to its advantages, such as multi-targets and low side effects. In this study, a mouse model of Dextran Sulfate Sodium (DSS)-induced acute colitis was established and the efficacy of Zhenqi Granule, a TCM preparation composed of the extractives from Astragali Radix and Fructus Ligustri Lucidi, was evaluated. The results showed that treatment with Zhenqi Granule prior to or post-DSS induction could alleviate the symptoms of colitis, including weight loss, diarrhea, hematochezia, colon length shortening, and pathological damage of colon tissues of the DSS-treated mice. Further, network pharmacology analysis showed that there were 98 common targets between the active components of Zhenqi Granule and the targets of UC, and the common targets were involved in the regulation of inflammatory signaling pathways. Our results showed that Zhenqi Granule had preventive and therapeutic effects on acute colitis in mice, and the mechanism may be that the active components of Zhenqi Granule participated in the regulation of inflammatory response. This study provided data reference for further exploring the mechanism of Zhenqi Granule and also provided potential treatment strategies for UC.

## 1. Introduction

Inflammatory bowel disease (IBD) is a group of chronic inflammatory diseases affecting the digestive tract, significantly impacting quality of life. The two main types of IBD are ulcerative colitis (UC) and Crohn’s Disease [1]. They both have complex and unclear causes and usually display similar symptoms but differ in affected parts of the digestive tract and the features of inflammation [1]. UC has a complex etiology and pathogenesis that is not fully understood [2]. Currently, the global incidence of UC is estimated to reach as high as 5 million cases and is still rising [3]. UC can occur at any age, with a predominant proportion of cases occurring between the ages of 20 and 40 [4]. Clinical symptoms of UC include weight loss, diarrhea, bloody stools, and abdominal pain [5]. The etiology of UC has not been completely identified but may include genetic background, environmental factors, immune dysregulation, gut microbial dysbiosis, oxidative stress, gut barrier dysfunction, etc. [6,7]. In UC, the intestinal barrier is destroyed, the immune system is activated, and the intestinal mucosa has an inflammatory response accompanied by increasing pro-inflammatory cytokines, such as TNF-α, IL-6, and IL-1β [7]. Moreover, frequent recurrence and a poor prognosis are also critical concerns of UC. In any case, drug intervention is the preferred method for the treatment of UC, in which aminosalicylic acid, glucocorticoids, antibiotics, immunosuppressive drugs, and anti-tumor necrosis factors have been used [8,9]. However, these drugs have limitations, including poor efficacy, high costs, and non-negligible side effects. For example, aminosalicylic acid could cause gastrointestinal reactions, liver damage, and occasionally allergies [10]; corticosteroids could cause osteoporosis [5]; and immunosuppressive drugs can weaken the body’s resistance, cause poor physique, and even cause other diseases [11]. Therefore, there is still a lack of drugs that can ideally cue UC [12]. Thus, it is worthwhile to investigate new and effective therapeutics.

Dextran Sulfate Sodium (DSS), a water-soluble sulfate polysaccharide, has been widely used to induce experimental UC models in mice [13]. Although the pathogenesis of DSS-induced UC is complex and not well clarified [14], it has been reported that DSS treatment could disrupt colonic epithelial cells in the basal crypt [15]. This causes pathological damage to intestinal tissues, which allows intestinal microorganisms and their metabolites to penetrate the epithelial layer and activate related signaling pathways, such as TLR4/TRIF/NF-κB, resulting in the secretion of pro-inflammatory cytokines [16]. In the DSS-induced mouse model of UC, clinical symptoms, including weight loss, diarrhea, bleeding, inflammatory infiltration, epithelial cell loss, and ulcer formation, were the hallmarks of UC [17,18].

Traditional Chinese Medicine (TCM) has the advantages of multiple components, multiple targets, and low side effects [19]. Zhenqi Granule is a TCM preparation made from the extractives of Astragali Radix (Huangqi) and Fructus Ligustri Lucidi (Nvzhenzi). This TCM formula has been reported to have immune modulation and anti-tumor activities and is clinically used for immunity improvement [20]. Modern pharmacological studies have shown that Astragali Radix has the effects of immunomodulatory, antiviral, hypoglycemic, diuretic, anti-aging, anti-oxidation, and anti-fatigue [21,22,23]. Fructus Ligustri Lucidi has been reported to have a variety of effects, including immune enhancement, hepatoprotective, anti-tumor, anti-osteoporosis, anti-inflammation, and anti-oxidant properties [24,25,26]. Moreover, each of the individual components of the formula, Astragali Radix and Fructus Ligustri Lucidi, has been shown to have a therapeutic effect in treating colitis, respectively. Astragalus polysaccharide, an extract of Astragali Radix, showed an anti-colitis effect in DSS-induced mice, which may function through systemic immune regulation [27,28,29,30]. Yu et al. reported that Fructus Ligustri Lucidi had therapeutic efficacy in murine models of experimental colitis by suppressing inflammation and rescuing dysbiosis [31]. However, so far, the efficacy of the combination of the two medicines has not been tested, and the underlying mechanism of how it exerts the therapeutic effect remains to be revealed.

Network pharmacology is an analytical method used to systematically study the interaction between drug components and multiple targets [32]. It is an important method used to analyze the molecular mechanism of complex drugs through the component–target–disease network [33]. The emergence of network pharmacology has broken the limitations of “the one-drug, one-target” research strategy [34]. TCM has various components and complex mechanisms of action and has long been in the stage of empirical medication and phenotypic analysis. The development of network pharmacology has promoted TCM from empirical medicine to modern pharmacological mechanism research and improved its recognition in the world [33].

In this study, we used the DSS-induced mouse model of colitis to explore the anti-colitis effect of Zhenqi Granule. The preventive and treatment effectiveness of Zhenqi Granule against colitis were evaluated by monitoring indicators, including body weight, diarrhea, bloody stool, colonic injury, and pro-inflammatory cytokines in colonic tissues. Moreover, network pharmacology was used to identify the interaction of the components of Zhenqi Granule with the key signaling pathways involved in the UC to analyze the possible mechanism of its action. This study is the first to explore the prevention and therapeutic effect of Zhenqi Granule on DSS-induced UC in mice and to predict its possible mechanism. Our study will be helpful in providing potential prevention and treatment strategies for UC.

## 2. Materials and Methods

### 2.1. Materials

Zhenqi Granule was purchased from HVSEN Biotech (Wuhan, China), which is extracted and concentrated from the decoction of Astragali Radix and Fructus Ligustri Lucidi made from Astragali Radix and Fructus Ligustri Lucidi. Each gram of Zhenqi Granule contains a minimum of 1 mg of Nuezhenoside and 0.1 mg of Astragaloside. DSS was purchased from MP Biomedicals (Irvine, CA, USA). 5-aminosalicylic acid (5-ASA), which was used as a positive drug against UC, was purchased from Sigma-Aldrich (Beijing, China) (L6511). ELISA kits of TNF-α (Cat#EK282), IL-6 (Cat#EK206), IL-1β (Cat#EK201B), and IL-17A (Cat#EK217) were purchased from Multi Sciences (Hangzhou, China).

### 2.2. Animals

Six- to eight-week female C57BL/6 mice were purchased from the Laboratory Animals Center of Huazhong Agricultural University and maintained at specific pathogen-free conditions at a 12 h light/dark cycle, a temperature of 24 ± 1 °C, and a relative humidity of 40~70%. Mice were provided free access to standard food and water and were acclimatized for 7 days before the start of the experiment. The animal experiment in this study strictly followed animal welfare and the Guide for the Care and Use of Laboratory Animals and was approved by the Laboratory Animals Ethics Committee of Huazhong Agricultural University. The approval number is HZAUMO-2023-0232.

### 2.3. Animal Treatment

A DSS-induced mouse colitis model was established as previously described [35]. Briefly, the DSS group mice received free drinking water containing 3% DSS (*w*/*v*) solution for 7 days, followed by normal drinking water, and the control group mice received normal drinking water. The mice were weighed daily, and 6 mice were dissected on days 3, 7, 8, 9, 11, and 13 in the DSS group, respectively. Six mice in the control group were euthanized on day 13. The stool was collected, and colon length and pro-inflammatory cytokines in the colon were measured.

To evaluate the preventive effect of Zhenqi Granule on DSS-induced mouse colitis, mice were divided into four groups (*n* = 6), which were (1) the control group, where mice received normal drinking water and oral gavage with 200 μL of drinking water for 10 days; (2) the DSS group, where mice were administered with 3% DSS solution for 7 days and oral gavage with 200 μL of drinking water for 10 days; (3) the DSS+ZQ-170 mg group, where mice were administered with 3% DSS for 7 days and oral gavage with the same volume of 170 mg/kg·bw of Zhenqi Granule for 10 days; and (4) the 5-ASA group, where mice were administered with 3% DSS for 7 days and oral gavage with the same volume of 100 mg/kg·bw of 5-ASA for 10 days. Body weight, bloody stools, and diarrhea were monitored daily. Mice were sacrificed and colon tissues were collected on day 10 of the experiment for detecting the colon length, pro-inflammatory cytokines and HE staining.

The therapeutic effect of Zhenqi Granule on DSS-induced mice colitis was evaluated as follows. Mice were divided into five groups (*n* = 10): the control group, the DSS group, the ZQ-170 mg group, the ZQ-340 mg group, and the 5-ASA group. The mice in the DSS group, ZQ-170 mg group, ZQ-340 mg group, and 5-ASA group were given drinking water containing 3% DSS solution for 7 days. Then, the mice in the ZQ-170 mg group, ZQ-340 mg group, and 5-ASA group were given 170 mg/kg·bw of Zhenqi Granule, 340 mg/kg·bw of Zhenqi Granule, and 100 mg/kg·bw of 5-ASA, respectively, by oral gavage for 10 days. Mice in the control group received normal drinking water. Body weight, bloody stools, and diarrhea were monitored daily. Mice were sacrificed and colon tissues were collected on day 17 of the experiment to detect the colon length and pro-inflammatory cytokines.

### 2.4. Disease Activity Index (DAI) Calculation

The mice were monitored daily for assessment of colitis according to DAI score composed of body weight loss score (0, none; 1 = 1~5%; 2 = 5~10%; 3 = 10~20%; 4 = over 20%), feces status score (0, normal; 1 = paste stools; 2 = loose stools; 3 = diarrhea; 4 = water stool), and bloody stools score (0, negative; 2 = bleeding; 4 = gross bleeding) [36].

### 2.5. Determination of the Levels of Pro-Inflammatory Cytokines in Colons

100 mg colon tissue was weighed and homogenized with 900 µL of phosphate buffer solution (PBS) for 15 min, followed by centrifugation at 5000× *g* for 10 min at 4 °C to obtain supernatant, stored at −80 °C. The concentration of pro-inflammatory cytokines including TNF-α, IL-6 and IL-1β in supernatant were measured using ELISA kits according to the manufacturer’s instructions.

### 2.6. Histological Analysis

The colon tissues were fixed in 10% formalin solution for 24 h and then dehydrated by successively soaking in different concentrations of ethanol ranging from 30% to 100%. The tissue was then washed with xylene followed by embedding in paraffin. Subsequently, 4 μm-thick sections of the colon tissues were cut and subjected to dewaxing with xylene and followed by hydration in ethanol solution from high concentration to low concentration. The sections were then stained with hematoxylin and eosin (HE). Finally, the stained sections were observed and assessed under a light microscope.

### 2.7. Network Pharmacological Analysis

Compounds in both Astragali Radix and Fructus Ligustri Lucidi were obtained from the TCMSP database (http://tcmspw.com/tcmsp.php, accessed on 7 March 2023), among which those that met the criterion of OB ≥ 30, DL ≥ 0.18 were taken as the active components. Targets of the activated components were analyzed in the TCMSP database and SwissTargetPrediction (http://www.swisstargetprediction.ch, accessed on 7 March 2023). Targets of UC were retrieved in the genecards database (http://www.genecards.org, accessed on 8 March 2023) [37]. Targets were transformed to corresponding gene names in the UniProt database (https://www.uniprot.org/, accessed on 17 March 2023). Their intersections were taken as the common targets. The common targets were used as the input to DAVID for GO and KEGG enrichment analysis.

### 2.8. Statistical Analysis

**Figure 1:**
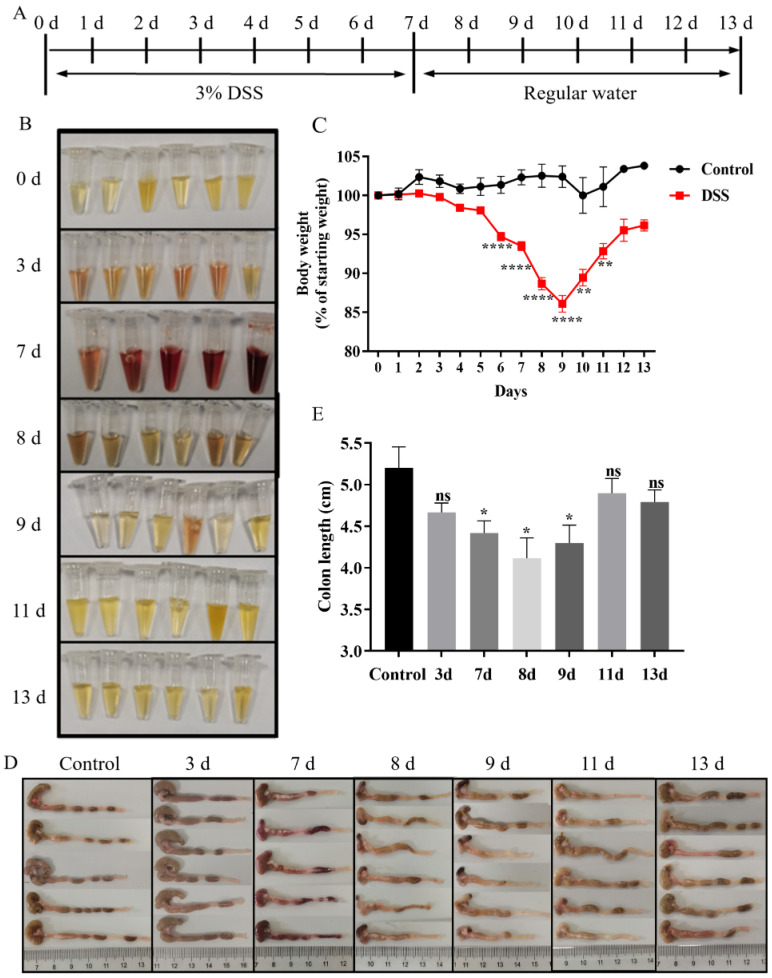
DSS-induced mouse colitis model. (**A**) Diagram of experimental procedure. (**B**) Mouse stool supernatant. Fresh mouse feces were collected and mixed with normal saline. The supernatant was obtained by centrifugation and then imaged. (**C**) Curve of mouse body weight. Six mice were included in each group, and the body weight was measured for every mouse every day. Student’s *t*-test was used for statistical analysis. ** *p* < 0.01 and **** *p* < 0.0001. (**D**) Image of the colon. The mice were euthanized, and the colon of each mouse was collected for imaging. (**E**) Statistics of colon length. The statistical difference in colon length was analyzed by one-way ANOVA. ns *p* > 0.05, * *p* < 0.05.

All statistical analyses were performed using GraphPad Prism 8 (GraphPad Software, San Diego, CA, USA). The unpaired *t*-test or one-way ANOVA was used to evaluate the difference in body weight loss, DAI data, colon length, and pro-inflammatory cytokine (the *t*-test was used in Figure 1C, and the one-way ANOVA was used for the other data). All data were presented as the mean ± standard errors of the mean (SEM). *p* < 0.05 was considered a significant difference. *, **, ***, and **** represent *p* < 0.05, *p* < 0.01, *p* < 0.001, and *p* < 0.0001, respectively.

## 3. Results

### 3.1. Establishment of the DSS-Induced Mouse Colitis Model

The mouse colitis model was established by giving drinking water containing 3% DSS solution for 7 days, and the clinical symptoms were recorded at different time points after DSS treatment (Figure 1A). It was shown that the mice developed bloody stools on day 3, with the most severe bloody stools on day 7. After stopping DSS treatment, bloody stools began to recover on day 8 and disappeared on day 9 (Figure 1B). The body weight of the mice started to decrease after drinking the DSS solution, which continued to decrease after the stop of DSS treatment, reaching a minimum weight on day 9 and then gradually recovering afterwards (Figure 1C). Colon length was significantly shortened during the period of day 7 to day 9 (Figure 1E). These suggested that the DSS-induced mouse colitis model was successfully established and there was a significant change in body weight, stools, and colon length.

### 3.2. Preventive Effects of Zhenqi Granule on DSS-Induced Mouse Colitis

**Figure 2:**
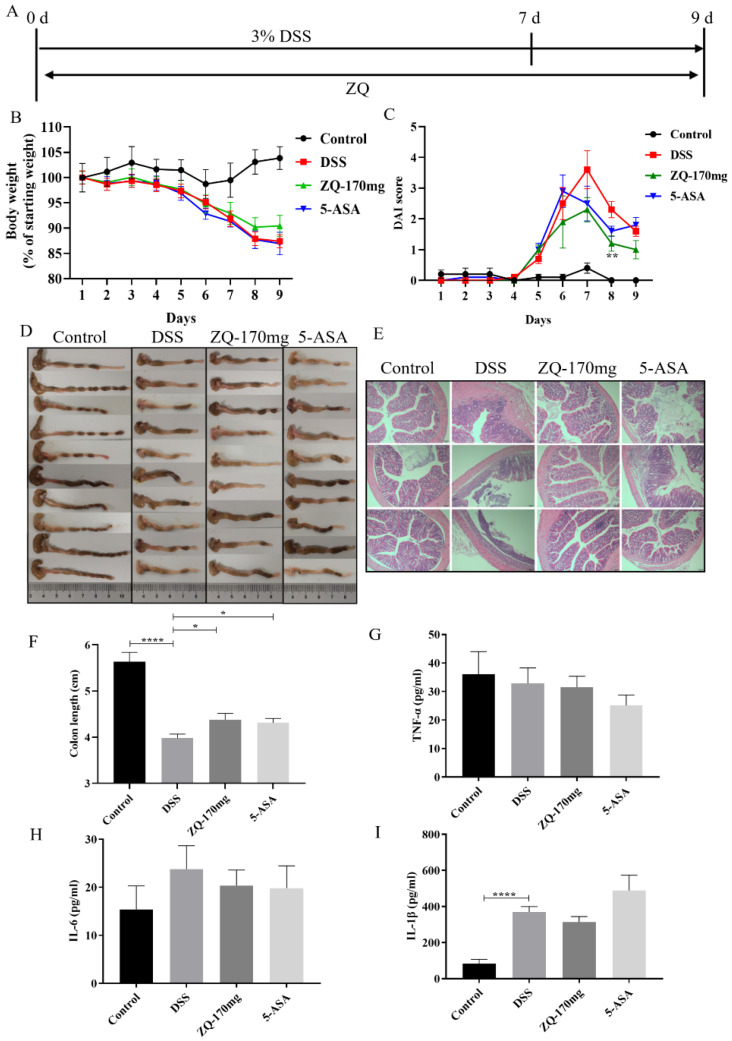
Zhenqi Granule pretreatment on DSS-induced mouse colitis. (**A**) Diagram of experimental procedure. (**B**) Curve of mouse body weight. Ten mice were included in each group, and the body weight was measured for every mouse every day. Student’s *t*-test was used for statistical analysis. (**C**) Disease activity index (DAI) score. DAI score is composed of body weight loss score, feces status score, and bloody stool score and was calculated as described in Section 2.4 in the Materials and Methods Section. ** *p* < 0.01. (**D**) Image of the colon. The mice were euthanized, and the colon of each mouse was collected for imaging. (**E**) HE staining of the colon. The colon tissues were collected, embedded in paraffin, sectioned, stained with hematoxylin and eosin (H&E), and examined under a light microscope. The bar is 100 μm. (**F**) Statistics of colon length. The statistical difference in colon length was analyzed by one-way ANOVA. (**G**–**I**) The levels of TNF-α, IL-6, IL-1β in colon tissues. Ten mice were included in each group. The colon tissues were collected and homogenized in normal saline. The supernatant was subjected to ELISA analysis. The statistical difference in colon length was analyzed by one-way ANOVA. * *p* < 0.05 and **** *p* < 0.0001.

To assess the preventive effect of Zhenqi Granule on DSS-induced mouse colitis, mice were treated with 3% DSS solution for 7 days, and at the same time, Zhenqi Granules were administered at the start of DSS treatment and lasted for 9 days (Figure 2A). As shown in Figure 2B, compared with the control group, the body weight of mice in the DSS group began to decrease on day 5. The body weight of mice in the ZQ-170 mg group was higher than that in the DSS group on day 8 and day 9; however, no significant difference was observed. As shown in Figure 2C, the DAI score of mice in the DSS group began to increase on day 5, reached a peak on day 7, and then gradually decreased. DAI scores of mice in the ZQ-170 mg group were lower than the DSS group and showed statistical significance on day 8 (Figure 2C). Moreover, the colon length of mice in the DSS group was significantly shorter compared with the control group, while the colon length of mice in the ZQ-170 mg and 5-ASA groups were significantly recovered compared with the DSS group (Figure 2F). The results of the HE staining of the colon tissues showed that the colon of the DSS group demonstrated significant pathological lesions, including thinning of the intestinal wall, reduction in crypt glands, and severe loss of intestinal epithelial cells (Figure 2E), while the pathological damages were significantly milder in the ZQ-170 mg group and 5-ASA group (Figure 2E). The levels of IL-6, IL-1β, and TNF-α in the colon tissues were then measured in each group. It was revealed that comparable levels of TNF-α, IL-6, and IL-1β were detected between the DSS group and the ZQ-170 mg group or the 5-ASA group (Figure 2G–I). Overall, Zhenqi Granule pretreatment could alleviate DSS-induced mouse colitis to a certain extent.

### 3.3. Therapeutic Effects of Zhenqi Granule on DSS-Induced Mouse Colitis

**Figure 3:**
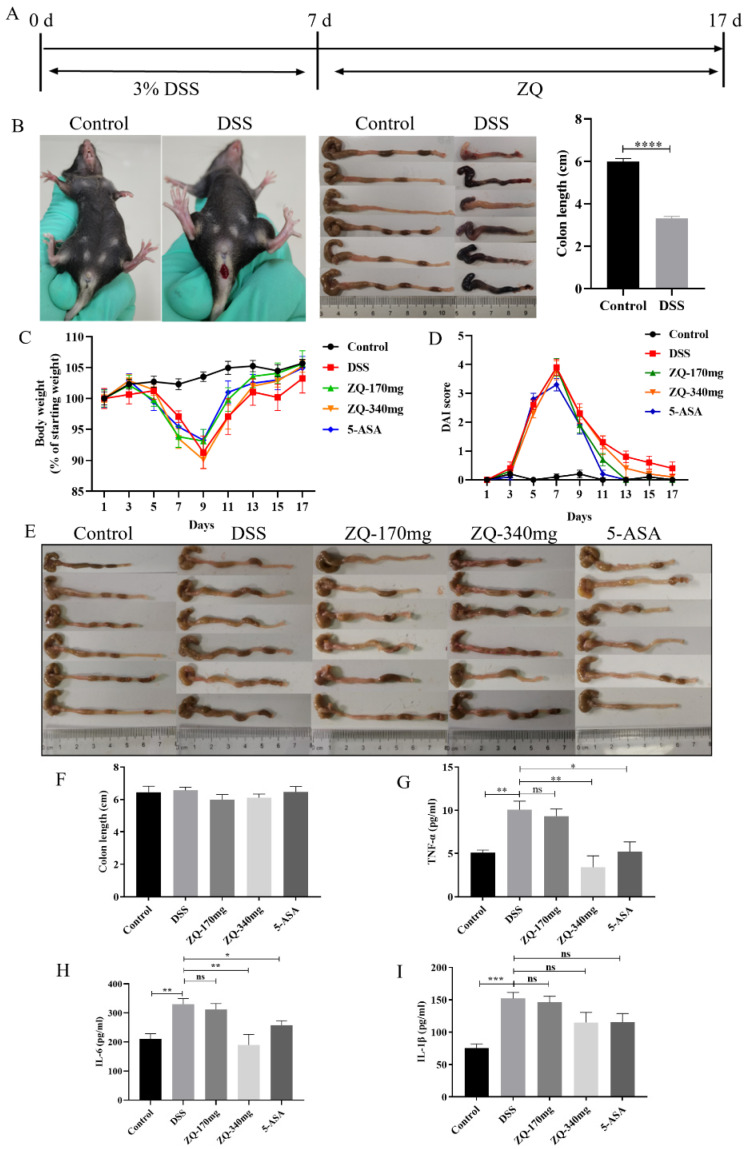
Zhenqi Granule post-treatment on DSS-induced mouse colitis. (**A**) Diagram of experimental procedure. (**B**) Blood stool and colon length of mice. **** *p* < 0.0001. (**C**) Curve of mouse body weight. Six mice were included in each group, and the body weight was measured for every mouse every day. (**D**) Disease activity index (DAI) score. DAI score is composed of body weight loss score, feces status score, and bloody stool score, which was calculated as described in Section 2.4 in the Materials and Methods Section. (**E**) Image of the colon. The mice were euthanized, and the colon of each mouse was collected for imaging. (**F**) Statistics of colon length. (**G**–**I**) The levels of TNF-α, IL-6, IL-1β in colon tissues. Ten mice were included in each group. The colon tissues were collected and homogenized in normal saline. The supernatant was subjected to ELISA analysis. The statistical difference in colon length was analyzed by one-way ANOVA. ns *p* > 0.05, * *p* < 0.05, ** *p* < 0.01, and *** *p* < 0.001.

Next, we tested whether Zhenqi Granule has a therapeutic effect on DSS-induced mouse colitis. A 3% DSS solution was given for 7 days to induce colitis and then Zhenqi Granule was administered afterwards, with two doses for 10 days (Figure 3A). As shown in Figure 3B, compared with the control group, mice in the DSS group showed bloody stools and significantly shortened colon lengths on day 7, indicating that a colitis model in mice was successfully established. It is shown in Figure 3C that the body weight of the mice in all groups started to increase on day 9. However, the body weight of the mice in the treatment groups recovered faster than those in the DSS group from day 11 to day 17, but the difference was not significant. As shown in Figure 3D, the DAI score of mice drinking the DSS solution began to increase on day 3, reached the highest peak on day 7, and then gradually decreased. The DAI score of the treatment group decreased faster than the DSS group from day 11 to day 17. That is, compared with the DSS group, the treatment group could alleviate weight loss and reduce the DAI of mice. On day 17, colons in each group had a normal appearance and no differences in length (Figure 3E,F), indicating that even the colon length of the DSS-treated mice had recovered. Figure 3G–I show that the administration of Zhenqi Granule at 340 mg/kg·bw decreased the levels of TNF-α, IL-6, and IL-1β compared with the DSS group. Therefore, Zhenqi Granule showed a therapeutic effect on DSS-induced mouse colitis.

### 3.4. Network Pharmacological Analysis

**Figure 4:**
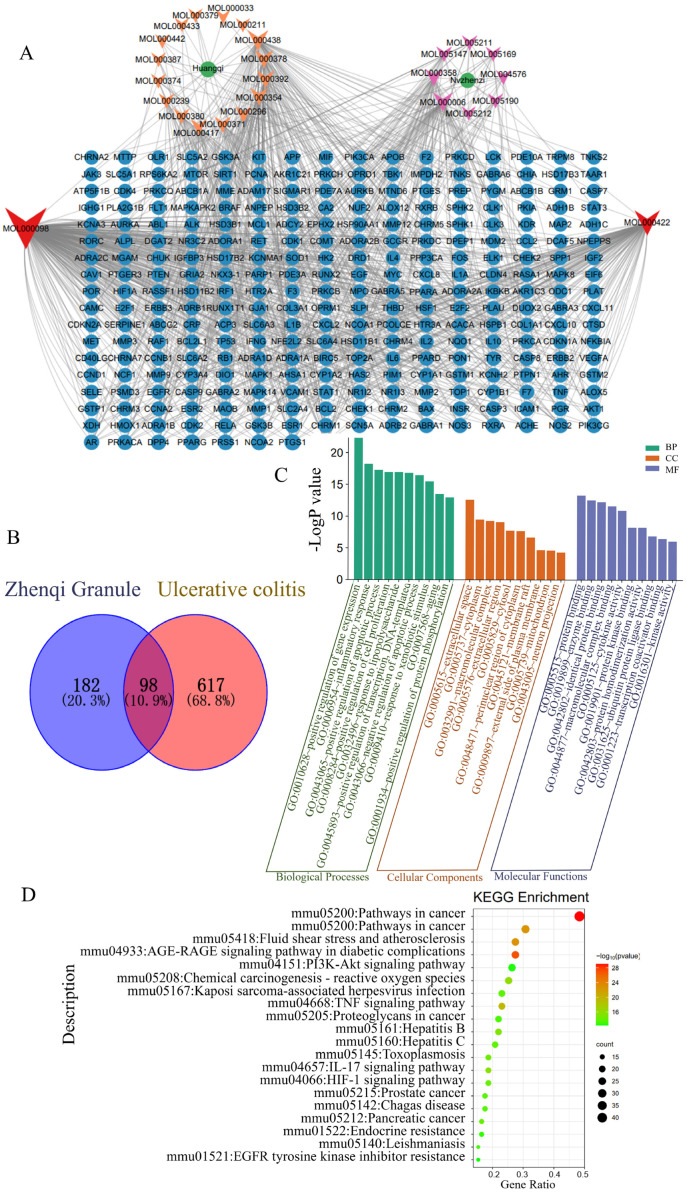
Network pharmacological analysis. (**A**) Compound–target network. The orange V-shaped symbol represents the active ingredients of Astragali Radix. The violet V-shaped symbol represents the active ingredients of Fructus Ligustri Lucidi. The red V-shaped symbol represents the common components of Astragali Radix and Fructus Ligustri Lucidi. The blue circle represents the protein targets. (**B**) Venn diagram of the common targets between Zhenqi Granule and ulcerative colitis-related protein targets. The targets of Zhenqi Granule were identified in the TCMSP and SwissTargetPrediction databases, and the targets of UC were retrieved from the GeneCards database. (**C**) GO and (**D**) KEGG enrichment analysis of the common targets. Enrichment analysis was conducted using the common targets as the input in DAVID.

Network pharmacological analysis was used to explore the potential mechanism of Zhenqi Granule against colitis. Active compounds in Astragali Radix and Fructus Ligustri Lucidi were analyzed by screened parameters, including oral bioavailability and drug-likeness, using the TCMSP database. The results showed that a total of 26 active compounds were identified (Figure 4A). The potential targets of these 26 active compounds were predicted using the TCMSP and SwissTargetPrediction databases, which showed a total of 280 proteins (Table S1). Also, a total of 715 proteins were predicted to be involved in UC by searching the GeneCards database. By analyzing the overlapping proteins, 98 common proteins were obtained, which were the targets of the active components acting on UC (Figure 4B and Table S2).

These common targets were subjected to the GO and KEGG enrichment analysis with DAVID. The GO enrichment analysis showed that 686 terms were significantly enriched (*p* < 0.05), including 546 in Biological Process (BP), 50 in Cellular Component (CC), and 90 in Molecular Function (MF) (Table S2). The top 20 items of them are shown in Figure 4C. The main BP items were the positive regulation of gene expression, the inflammatory response, the positive regulation of the apoptotic process, the positive regulation of cell proliferation, the response to lipopolysaccharide, etc. The main CC items were extracellular space, cytoplasm, macromolecular complex, extracellular region, etc. The MF items were protein binding, enzyme binding, macromolecular complex binding, cytokine activity, protein kinase binding, etc. The KEGG enrichment analysis showed that 135 significant pathways (*p* < 0.05) were enriched (Table S3). The top 20 items involving 135 signaling pathways were identified and shown in Figure 4D. These signaling pathways were related to cancer, inflammation, apoptosis, immunity, oxidative stress, necrosis, etc. Among them, inflammation-related signaling pathways were the main ones, including the TNF-α signaling pathway, the IL-17 signaling pathway, the toll-like receptor signaling pathway, the NF-κB signaling pathway, inflammatory bowel disease, etc. These indicated that the regulation of inflammatory signaling pathways was one of the important mechanisms of Zhenqi Granule.

## 4. Discussion

UC is characterized by progressive, recurrent, or remitting intestinal disease that seriously affects the quality of life of millions of people worldwide [9,38]. The incidence of UC is increasing worldwide and has an unsatisfactory cure rate [39]. TCM is a good choice for UC treatment due to its low side effects and multi-targets. In this study, a mouse model of DSS-induced colitis was applied to mimic UC, and the efficacy of Zhenqi Granule against colitis was investigated in this model with two methods of drug administration. Combined with network pharmacology analysis, which shed some light on the underlying mechanisms, our study showed the potential efficacy of Zhenqi Granule against UC, providing alternative options for treating such a complicated inflammatory disease.

Exploring the prevention of UC is of great significance. As we all know, one of the problems of UC is that it has a high recurrence rate [3,39]. The cause of IBD recurrence is uncertain, which is closely related to perpetuating inflammation [40], diet [41,42], genes [43], mindset [44], previous treatment methods, course and extent of the disease [3], etc. Among the common drugs for the treatment of UC, 5-ASA has no effects in severe UC [3], corticosteroids are not suitable for maintenance therapy, the long-term use of immunosuppressive agents can cause serious complications [45], biological therapies have high cost and potential adverse events [46,47], etc. Therefore, pharmacologic prophylaxis should be a better strategy, especially for people with genetic susceptibility. In addition, UC is a disease that is prone to recurrence [48]. In the calm period of the disease, it is a good strategy to use TCMs to reduce the recurrence rates of UC in the future.

To test the preventive efficacy against UC, Zhenqi Granule was pretreated, and then DSS was administrated. In the DSS-induced mouse model of UC, colon shortening and inflammations are the key indicators. Our results showed that Zhenqi Granule pretreatment significantly alleviated the shortening of the colon (Figure 2D,F). However, the colon length was not recovered to that of the control group. This indicates that Zhenqi Granule only showed preventive efficacy to some extent. Moreover, in this experiment, only one dose (170 mg/kg·bw) was used. This is because in our previous work studying the anti-inflammatory effect using a lipopolysaccharide-induced mouse model, 170 mg/kg·bw and 340 mg/kg·bw both showed anti-inflammatory efficacy (data to be published). Therefore, the duration and dose of the administration of Zhenqi Granule against UC need further optimization.

Treating patients with UC remains challenging, despite a range of treatment drugs, such as 5-ASA, corticosteroids, thiopurines, methotrexate, cyclosporine, tacrolimus, TNF-α antagonists, vedolizumab, tofacitinib, and ustekinumab [47,49]. However, the remission rate of UC is still far from optimal [50]. Proctocolectomy is required in 10–20% of patients [3], and the 10-year cumulative risk of relapse is 70–80% [48]. Efforts to expand drug development for the treatment of UC should be continued. In this study, Zhenqi Granule treatment post-DSS induction can alleviate weight loss and the DAI score of mice to a certain extent and significantly alleviate the levels of pro-inflammatory cytokines in colon tissues. Inflammation is an important feature of UC [51,52], and Zhenqi Granule can significantly inhibit colonic pro-inflammatory cytokines in DSS-induced mouse colitis. This indicates that Zhenqi Granule has great potential in the treatment of UC in the future.

Network pharmacology has been developed rapidly in the field of TCM due to its advantages of systematic and comprehensive analysis of the action mechanism of multi-component drugs [34]. In order to further explore the anti-colitis mechanism of Zhenqi Granule, network pharmacology analysis was performed. The results of enrichment analysis showed that these targets are mainly involved in inflammation-related signaling pathways. Many of the active ingredients of Zhenqi Granule, including quercetin, kaempferol, (3R)-3-(2-hydroxy-3,4-dimethoxyphenyl)chroman-7-ol, luteolin, 7-O-methylisomucronulatol, formononetin, isorhamnetin, beta-sitosterol, hederagenin, and 3,9-di-O-methylnissolin, have been reported to have anti-inflammatory effects [53,54,55,56,57,58,59]. The basic principle of DSS-induced acute colitis in mice is that DSS destroys the intestinal epithelium [60]. As a result, intestinal microorganisms, lipopolysaccharides, and other harmful substances penetrate the epithelial layer and activate the inflammatory pathway mediated by NF-κB, TNF, etc., resulting in the secretion of a large amount of pro-inflammatory cytokines [61,62]. More evidence suggests that the main pathogenesis of UC is excessive inflammatory response [40]. The animal experiments in this study have also shown that Zhenqi Granule treatment can significantly inhibit pro-inflammatory cytokines. We predicted that the potential mechanisms of Zhenqi Granule against UC may be involved in the regulation of inflammatory response.

Utilizing network pharmacology analysis, the investigation of the anti-inflammatory mechanism of Astragali Radix in a recent study showed that the active compounds of Astragali Radix (quercetin, kaempferol, 7-O-methylisomucronulatol, formononetin, etc.) were involved in regulating key targets (TNF, TLR4, IL10, etc.) on inflammatory signaling pathways (IL-17 signaling pathway, TNF signaling, etc.) [63]. An et al. used network pharmacological analysis to conclude that the active components of Astragalus were involved in inhibiting IKKβ on the NF-κB signaling pathway [64]. Hu et al. used network pharmacological analysis to find that the active compounds of Jinfeng pills (luteolin, kaempferol, quercetin, etc.) were involved in the regulation of IL-17, Th17 cell differentiation, the TNF signaling pathway, and the MAPK signaling pathway, suppressing the phosphorylation of ERK1/2 and protein levels of IL-17A and IL-6 [65]. These results indicated that the active compounds of Astragali Radix and Fructus Ligustri Lucidi were involved in regulating key targets in inflammatory pathways to achieve anti-inflammatory effects, which was consistent with the network pharmacological analysis in this study. However, it has to be pointed out that experimental validation of the underlying mechanism is still needed.

## 5. Conclusions

Preventive administration of Zhenqi Granule demonstrated a mitigating effect on symptoms induced by DSS in mice, while therapeutic administration exhibited a notable reduction in inflammatory mediators. Network pharmacological analysis predicted that the potential mechanism of the main components of Zhenqi Granule was involved in regulating inflammatory signaling pathways. This study serves as a foundational exploration of the therapeutic properties of Zhenqi Granule, presenting promising prospects for the advancement of innovative treatment of UC.

## Data Availability

The data for this study can be obtained from the corresponding author upon reasonable request.

## Supplementary Materials

The following supporting information can be downloaded at https://www.mdpi.com/article/10.3390/biology13060427/s1, Table S1: Compound-target network. Table S2: Common targets. Table S3: GO enrichment. Table S4: KEGG enrichment.
